# An improved spider optimization algorithm coordinated by pheromones

**DOI:** 10.1038/s41598-022-09800-x

**Published:** 2022-04-08

**Authors:** Siling Feng, Yue Hu, Yinjie Chen, Mengxing Huang

**Affiliations:** 1 School of Information and Communication Engineering, Hainan University, No. 58 Renmin Avenue, Haikou, 570028 Hainan China; 2 School of Science, Hainan University, No. 58 Renmin Avenue, Haikou, 570028 Hainan China; 3 State Key Laboratory of Marine Resource Utilization in the South China Sea, Hainan University, No. 58 Renmin Avenue, Haikou, 570028 Hainan China

**Keywords:** Computational science, Computer science

## Abstract

Swarm intelligence algorithm is an important evolutionary computation method that optimizes the objective function by imitating the behaviors of various organisms in nature. A two-stage swarm intelligence algorithm named spider pheromone coordination algorithm (SPC) is proposed in this paper. SPC tries to explore as many feasible solutions as possible on the cobweb at the positioning stage. It simulates the release and reception of different pheromones between spiders at the hunting stage, and then spiders move towards prey under the co-action of winds and pheromones. Different from the existing algorithms, SPC simulates the process that spiders accomplish intra-species communications through different pheromones and considers the impact on spider wind movement. A large number of typical benchmark functions are used in comparative numerical experiments to verify the performances of SPC. Experiments are made to compare SPC with a series of swarm intelligence algorithms, showing that SPC has higher convergence accuracy and stronger global searchability, effectively keeping the diversity of feasible solutions.

## Introduction

Swarm intelligence algorithm is the intersection of bionic science and computer science^1^, it constructs solving strategies for computation problem or distributed problems by abstracting social behaviors such as mutual cooperation, intra-species competition, and intra-species communication between biological individuals^2,3^. As new evolutionary computation technologies, many swarm intelligence algorithms have been proposed by researchers. The basic idea is to simulate the behaviors of natural biological groups to construct stochastic optimization algorithms. The biological mechanisms of algorithms are different. Artificial Bee Colony (ABC)^4^ was proposed by Karaboga in 2005 by simulating the honey gathering activities of bee colonies. Ant Colony Optimization (ACO)^5^ adopts a positive feedback mechanism to complete the transmission of information between populations, which is the most significant characteristic different from other bionic algorithms. Grey Wolf Optimization (GWO)^6^ originates from the simulation of predation behaviors of the gray wolf group and realizes the optimization through the process of wolf pack tracking, surrounding, chasing, and attacking prey. Harris Hawk Optimization (HHO)^7^ was inspired by the cooperative behavior and chasing style of harris hawks in nature. Monarch Butterfly Optimization (MBO)^8^ simulates the migration behavior of monarch butterflies. Particle Swarm Optimization (PSO)^9^ has a simple mechanism, which is designed by simulating the hunting behaviors of birds. Besides the works mentioned above, there are many excellent works in this area, such as Earthworm optimization Algorithm (EWA)^10^, Elephant Herding Optimization (EHO)^11^, Moth Search (MS) algorithm^12^, Slime Mould Algorithm (SMA)^13^, Hunger Games Search (HGS)^14^, Runge Kutta optimizer (RUN)^15^ and so on.

There have two existing swarm intelligence algorithms that originated from spiders. Social Spider Optimization (SSO)^16,17^ was proposed by Cuevas through simulating the behaviors of social spiders such as mutual predation, exchanging information, reproduction, and generations of offspring. According to the female and male, spiders are classified into two categories, and the optimizing process is based on different criteria. Social Spider algorithm (SSA)^18,19^ originates from the natural phenomenon that spiders spread signals to attract other spiders to go foraging after discovering food. In SSA, when a spider finds its prey, it produces a strong vibration to attract the remaining spiders to move to it. Eventually, most of the spiders in the population will gather around the prey. However, the above algorithms generally have some defects such as slow speed of convergences, low accuracy of calculations, and low success rate of high-dimensional functions, as well as easy to fall into local optimization and so on^20,21^. There is also a contradiction between the diversity of populations and the rate of convergences of the algorithms^22,23^.

In order to solve the above problems, this paper proposes a spider pheromone coordination algorithm (SPC) according to the cooperative hunting behaviors of spiders and the biological characteristics that spiders have different responses to different kinds of pheromones. SPC is divided into two stages. The positioning stage aims to explore the whole feasible solution space as much as possible in order to find the outstanding solutions and the horrible solutions that reflect the global information. SPC gives the cobweb a certain memory so that it can selectively retain pheromones to guide the movements of spiders and make the movements of spiders difficult to fall into local optimization and avoid the premature convergence of the algorithm. At the hunting stage, spiders in SPC can release and distinguish different pheromones, and the memory of artificial cobwebs in SPC can store the information of extreme solutions. The spider’s movements are influenced by the wind and the pheromones that are distributed across the cobweb and eventually move closer to its prey. A large number of typical benchmark functions are used in the comparative numerical experiments to verify the performances of SPC. Compared with PSO algorithm^24^, ABC algorithm^25^, SSO algorithm^17^, SSA algorithm^18^, GWO algorithm^6^ and ACO algorithm^26^, MBO^8^, and HHO^7^. SPC has higher convergence accuracy and stronger global search ability, effectively keeping the diversity of feasible solutions.

The remainder of this paper is organized as follows. The biological characteristics of spiders are introduced in “Biological fundamentals” section. SPC is illustrated by simulating the effects of winds on the movements of spiders and the cooperative hunting behaviors of social spiders using pheromones and the cobweb in “Spider pheromone coordination algorithm” section. The differences between SPC and other algorithms are also described in “Spider pheromone coordination algorithm” section. The comparisons between SPC and other algorithms and the experimental results are performed and analyzed in “Experiment and result” section. The conclusions and future works are put forward in “Conclusion” section.

## Biological fundamentals

Pheromones are chemicals that interact within a population of organisms and can carry specific information. They can affect the behaviors of animals and have both stimulating and inhibiting effects^27,28^. Some literature reported the presence of social pheromones in social groups to coordinate social life of groups^29,30^. Silk of spiders is not only the effective predation tool for spider groups but also a good carrier of pheromones. A large number of studies have confirmed that spiders can use pheromones remaining on silk of spiders or released into the air to carry out inter-specific communications to realize mutual cooperative behaviors. That is to say, silk of spiders can carry different pheromones and the proportion of pheromones on the cobweb in different periods is different^31^.

Pheromones can be divided into aggregation pheromones, alarm pheromones, trail pheromones and mark pheromones. Aggregation pheromones cause other individuals of the same species to move to the site of release source, thus forming an aggregation of population. Alarm pheromone has a positive feedback effect that can cause other people in the population to sound an alarm or flee. Moreover, the tracking pheromone has a negative feedback effect that can tell the traces of a person to other people in the same species and instruct other people in the population to transfer to the release source^28,32–34^.

Spiders can use their silk to weave cobwebs. The silk of spiders and cobwebs are cornerstone of social activities of spiders^35^ which can prevent excessive dispersion of spiders in the population and play an essential role in cooperation^36^. This shows the importance of cobwebs to social spiders, indicating that social spiders live on a cobweb most of their time. Spiders in SPC are supposed to be unable to leave the cobweb and can only move on it.

Based on biological facts, the heavier a spider is, the less the winds affect its movement. Conversely, the lighter a spider is, the more the winds affect its movement. Apparently, the number of prey caught by a spider is proportional to its own weight. Therefore, at the positioning stage of SPC, the effects of winds on movements of spiders are relatively large, and at the hunting stage of SPC, the effects of wind on movements of spiders will decrease. It should be noted that in SPC algorithm, the winds directly affect the movements of spiders rather than the spread of pheromones.

## Spider pheromone coordination algorithm

SPC simulates the movements of social spiders which use pheromones for cooperative predation. In SPC, the optimization space of the optimization problem is abstracted into a multi-dimensional cobweb on which all social spiders live. Every location on the cobweb represents a feasible solution to the optimization problem. The locations on the cobweb form a corresponding one-to-one relationship with feasible solutions to the optimization problem. The optimal solution of this optimizing problem can be equivalently converted to finding an optimal location on the cobweb, the location of which depends on the size of prey that determines the objective function of the optimization problem. The value of objective function is the fitness value of spiders. Importantly, spiders may get out of the cobweb during random movements. However, spiders only can move freely on the web, because the locations outside cobweb represent unworkable solutions to the optimization problem. There are many methods to deal with boundary constraints in the previous literature, among which random method, absorption method and reflection method are the most widely used^37^. In this paper, stochastic method is used to deal with constraints.

Releasing and receiving pheromones are a form of communication within the population of spiders. When a spider begins to move, it receives pheromones distributed on the web, combining the winds, then it will choose which direction to move. When all spiders reach new locations, they start to release pheromones whose concentrations and types depend on the values of the objective function, that is, the fitness value of the spiders at these locations. Cobwebs are the transmission media of spiders’ pheromones. The pheromones released by one spider will be received by all the other spiders in the population and affect movements of the rest of spiders. A global sharing information model is established by spiders sharing their own information indirectly through cobwebs and pheromones.

After enough movements, the spiders will master the global information roughly, which can make a significant contribution to the subsequent optimization. SPC gives the cobweb a specific intelligence memory to record the outstanding solutions and the horrible solutions produced in every iteration at the first stage. After the positioning stage is completed, all the pheromones on the cobweb will be cleared entirely. In order to continuously guide the subsequent optimization process, the more representative extreme solutions will be selected from the solutions recorded on the cobweb, and the pheromones in their corresponding locations are updated to be marked pheromones or trail pheromones.

It should be noted that only mark pheromones and trail pheromones have persistent effects. In contrast, aggregation pheromones and alarm pheromones can only affect the movements of spiders in the next iteration. When the next iteration is completed, the original aggregation pheromones and alarm pheromones are cleared, spiders will release new aggregation pheromones and alarm pheromones.

### Initializing population

The convergence speed of the SPC algorithm and the quality of the solutions are affected by the initialization of population. The initial population with good diversity is beneficial to improving the algorithm’s optimization performance. In order to make the initial population have a certain degree of dispersion, the random method is used to initialize the population. This ensures the global search efficiency of the algorithm, the operability of the algorithm, and the diversity of individuals in the initial population.

The population size of spiders is invariable, which is denoted as *N*. The location of every spider in the *D* - dimensional space can be described as: ${{x}_{i}}=\left( {{x}_{i1}},{{x}_{i2}},\ldots ,{{x}_{id}},\ldots ,{{x}_{iD}} \right)$ , a feasible solution of optimization problem can be evaluated by the corresponding objective function ${{f}_{i}}$ that is the evaluation of the size of prey of the location. According to the value ${{f}_{i}}$ , the fitness value of location ${{x}_{i}}$ and spider *i* can be measured. By comparing the current location of each spider in initial population, the optimal location and the worst location in initial population were obtained, and the value of the objective function of the optimal location in the initial population was recorded as *M*.

### Positioning stage

The hunting behaviors of social spiders can be described as the movements of spiders towards the locations of prey. Spiders receive and distinguish pheromones on the web to determine the potential directions of prey. The natural behaviors of spiders are used to optimize the solution space in SPC algorithm.

**Figure 1:**
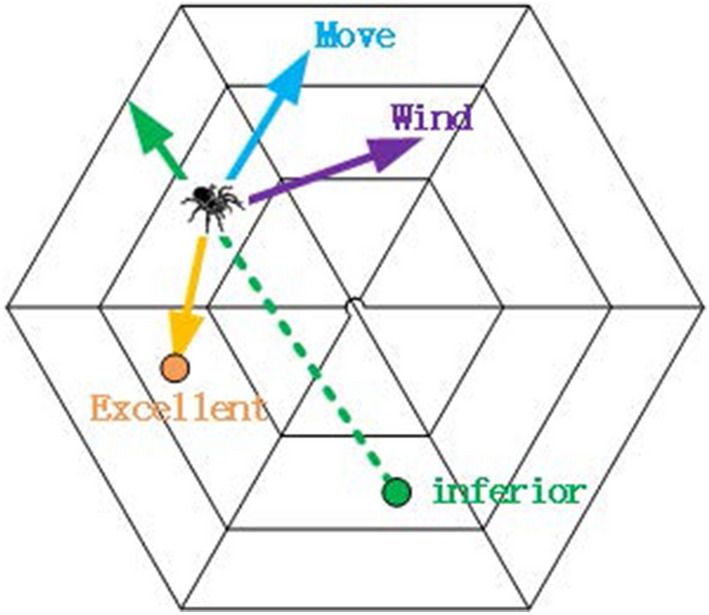
Positioning stage.

In the positioning stage, spiders only release aggregation pheromones and alarm pheromones. The cobweb records the locations of the terrible solution and the excellent solution produced by each iteration. While all spiders move to the new location, the pheromone is updated. The process in the positioning stage is shown in Fig. 1.

The locations of spiders are affected by the types and concentrations of pheromones released by other spiders. We take the value *M* of the optimal objective function in the initial population as the criterion. If the fitness value ${{f}_{j}}$ of spider *j* is better than *M* , Spider *j* releases aggregation pheromones that attract other spiders of the population to move closer to the location ${{x}_{j}}$. The greater the absolute value of the difference between ${{f}_{j}}$ and *M* , the higher the concentration of aggregation pheromones released by spider *j*. On the contrary, if the fitness value ${{f}_{j}}$ of spider *j* is less than *M* , Spider *j* releases alarm pheromones, telling other spiders to move away from the location ${{x}_{j}}$. The greater the absolute value of the difference between ${{f}_{j}}$ and *M* , the higher the concentration of the alarm pheromones released by spider*j*. That is, the type of pheromones released by spider *j* is determined by the relationship between its corresponding fitness value ${{f}_{j}}$ and the size of *M* , and the concentration of pheromones released is proportional to the absolute value of the difference between ${{f}_{j}}$ and *M*.

The effect ${{z}_{ji}}$ of spider *j* on spider *i* can be quantitatively described by the following formula:

1 $$\begin{aligned} {{z}_{ji}}=\left( {{f}_{j}}-M \right) \times \left( {{x}_{j}}-{{x}_{i}} \right) \end{aligned}$$

${{x}_{j}}$ and ${{x}_{i}}$ represent the locations of spider *j* and spider *i *respectively, ${{f}_{j}}$ is the fitness value of spider *j*.

The movements of spider *i* are affected by all the pheromones on the web. Then, the effect ${{Z}_{i}}$ of other spiders in the population on the movements of spider *i* , which the following formula can quantitatively give:

2 $$\begin{aligned} {{Z}_{i}}=\sum \limits _{j=1}^{N}{{{z}_{ji}}}=\sum \limits _{j=1}^{N}{\left( {{f}_{j}}-M \right) }\times \left( {{x}_{j}}-{{x}_{i}} \right) \end{aligned}$$

It should be noted that aggregation pheromones and alarm pheromones are highly volatile. In other words, the aggregation pheromones and alarm pheromones produced by every spider in every iteration only affect the movements of the spiders in the current iteration. They do not affect the movements of the spiders in the subsequent iteration. The aggregation pheromones and alarm pheromones indicated by formula 1 do not decay with distance or dissipate with time. Nevertheless, they will be updated at the end of each iteration.

### Movements of spiders at positioning stage

The movements of spiders in nature are affected not only by the pheromones released by other spiders but also by the winds to a large extent. The speeds and directions of natural winds are difficult to be predicted. In order to facilitate operation, we set the wind as *D*-dimensional random vector *wind* , which can be perceived by all spiders, is updated at the beginning of every iteration. The *wind* is given by the following formula:

3 $$\begin{aligned} wind=\left( \frac{2\times ub}{{{c}_{w}}} \right) \times random-ub/{{c}_{w}} \end{aligned}$$

*ub* is *D*-dimensional vectors, and it represents the upper bound of *D*-dimensional optimizing space, ${{c}_{w}}$ is a constant, *random* is a *D*-dimensional random vector in the range of 0 to 1. The effect ${{W}_{i}}$ of winds on spider *i* can be quantitatively described by the following formula:

4 $$\begin{aligned} {{W}_{i}}=wind\times \left( \frac{{{f}_{i}}}{{{c}_{f}}}-M \right) \end{aligned}$$

${{f}_{i}}$ is the fitness value of the spider *i* , and *M* is the best fitness value of the initial population. ${{c}_{f}}$ is a constant.

The moving step of a spider is described as *r* , it is a *D*-dimensional vector. It decreases with the increase of the number of iterations, which can be quantitatively described by the following formula:

5 $$\begin{aligned} r=\left( \frac{ub-lb}{{{c}_{r}}} \right) \times \cos \left( \frac{t\times \pi }{{{c}_{r}}\times I{{t}_{p}}} \right) \end{aligned}$$

*ub* and *lb* are *D*-dimensional vectors, representing the boundary value of the cobweb in each dimension. *ub *represents the upper bound of *D*-dimensional optimizing space, and *lb* represents the lower bound of *D*-dimensional optimizing space. ${{c}_{r}}$ is a constant. *t *represents the number of iterations at the positioning stage. $I{{t}_{p}}$ is the maximum number of iterations.

It should be noted that, in every iteration, the moving step *r* of the spiders in the population is the same. That is, spiders have the same ability to move. It also reflects the equality between spiders. The movements of spiders are realized by superposition of the current location vector and the moving vector, and are restricted by the moving step *r*. The moving vector depends on the winds and pheromones distributed on the web. Because spiders cannot fly, the locations of spiders do not change dramatically. That is, the starting location of every movement of the spider *i* is related to previous locations. The inertia weight ${{c}_{m1}}$ is added, which determines the effect of the current locations of the spider on the movement of the spider. Thus it plays the role of balancing the global optimization and local optimization of the algorithm.

The location ${{x}_{i}}$ of spider $i\left( i=1,2,\ldots ,N \right)$ is updated as follows:

6 $$\begin{aligned} {{x}_{i}}={{x}_{i}}/{{c}_{m1}}+{{Z}_{{{0}_{i}}}}\times r+wind\times \left( \frac{{{f}_{i}}}{{{c}_{f}}}\text {-}M \right) \end{aligned}$$

${{Z}_{{{0}_{i}}}}$ is the unit vector of ${{Z}_{i}}$. ${{c}_{m1}}$ is the inertial weight and we set it as a constant.

After all spiders have moved to the new locations, they start releasing pheromones according to the value of the objective function of the new locations to update the pheromones on the web. The pheromones updated in the *k* th iteration could only affect the movements of spiders in the $(k+1)$ th iteration.

The more preys in this location are, the more concentration of aggregation pheromones released by spiders, and the spider will move towards the location in the next iteration. On the contrary, the fewer preys in this location are, the more concentration of alarm pheromones released by spiders, and the spider will move further away from the location in the next iteration. Cobwebs record the horrible solution and the outstanding solution generated by every iteration. In this way, the positive and negative feedback mechanism for realizing the interactions of spiders is established. The reasonable solutions play the role of positive feedback regulation, while the bad solutions play the role of negative feedback regulation.

If the spiders exceed the boundary of cobweb in random movements, the stochastic method is used to deal with the situation.

### Selections of Cobweb

In SPC, the cobweb is given a certain intelligent memory, and the cobweb records the locations of the outstanding solution and the horrible solution produced by every iteration. At the end of the positioning stage, we can obtain $I{{t}_{p}}$ outstanding solutions and $I{{t}_{p}}$ horrible solutions. These extreme solutions are sorted by fitness values from the largest to the smallest. The *Y* feasible solutions with the largest fitness value are selected, and their locations and corresponding values of the objective function are recorded. The pheromones at the *Y* feasible solutions locations are updated to be mark pheromones, and their concentrations are determined by the value of the objective function at the corresponding locations. Similarly, *Y* feasible solutions with the minimum fitness values were selected, and their locations and corresponding values of the objective function were recorded. The pheromones at the locations with the *Y* feasible solutions are updated to be the trail pheromones. Their concentration is determined by the values of the objective function at the corresponding locations. The coordinates of the *Y* extremely good solutions are ${{g}_{s}}=\left( {{g}_{s1}},{{g}_{s2}},\ldots ,{{g}_{sD}} \right)$, where *D* are the dimensions of the optimizing space and $s=1,2,\ldots ,Y$.$f{{g}_{s}}$ is the value of its corresponding objective function. Accordingly, the coordinates of the *Y*extremely bad solutions are ${{b}_{s}}=\left( {{b}_{s1}},{{b}_{s2}},\ldots ,{{b}_{sD}} \right)$, where *D* are the dimensions of the optimizing space and $s=1,2,\ldots ,Y$.$f{{b}_{s}}$ represents the value of its corresponding objective function. It should be noted that, while the characteristics of aggregation pheromones and alarm pheromones are updated at the end of every iteration, mark pheromones and trail pheromones have continuous effects and can continuously guide the movement of spiders at the hunting stage of SPC.

Since the outstanding historical solutions and the horrible historical solutions are aimed at the global optimizing space, the 2*Y* extreme solutions reflect the global information. In order to strengthen the guiding role of the extreme solutions in the subsequent iterations of the spiders, the effect of other non-extreme solutions on the subsequent movements of spiders should be avoided. Therefore, only the pheromones at the locations representing the 2*Y* extreme solutions are updated. And the pheromones at the rest of the locations are all cleared to zero. The pheromones on the web can be received by all spiders at the hunting stage, and every spider can know the distribution of the extreme solutions. Thus, a global mechanism of communications for sharing information is formed.

### Hunting stage

At the hunting stage of SPC, the trail pheromones at ${{g}_{s}}$ will guide the surrounding spiders towards ${{g}_{s}}$. The mark pheromones at ${{b}_{s}}$ will guide the surrounding spiders to move away from ${{b}_{s}}$ ,where $s=1,2,\ldots ,Y$. Such a positive and negative feedback mechanism increases the possibility that the population of spiders will find better solutions.

At the hunting stage, there are three factors that affect the movements of spiders: one is the mark pheromones and trail pheromones stored on the web, the other is the aggregation pheromones and alarm pheromones produced by the population of spiders, and the third is the winds.

### Guidance of Cobweb

In order to measure the effectiveness of the 2*Y* extreme solutions on movements of spiders at the hunting stage, it is necessary to determine the influence space of mark pheromones and trail pheromones. We define a *D*-dimensional vector *R* , when spider *i* is in the *D*-dimensional solution space with *R* radius centered on an extreme solution, which means that the extreme solution will regulate and guide the movement of spider *i* .If spider *i* is not in this *D*-dimensional solution space, this extreme solution does not regulate and guide the movement of spider *i*. If spider *i* enters the *D*-dimensional solution space where the central location is trail pheromones, it will move towards the center. On the contrary, if spider *i* enters the *D*-dimensional solution space where the central location is marked pheromones, it will move away from the center.

**Figure 2:**
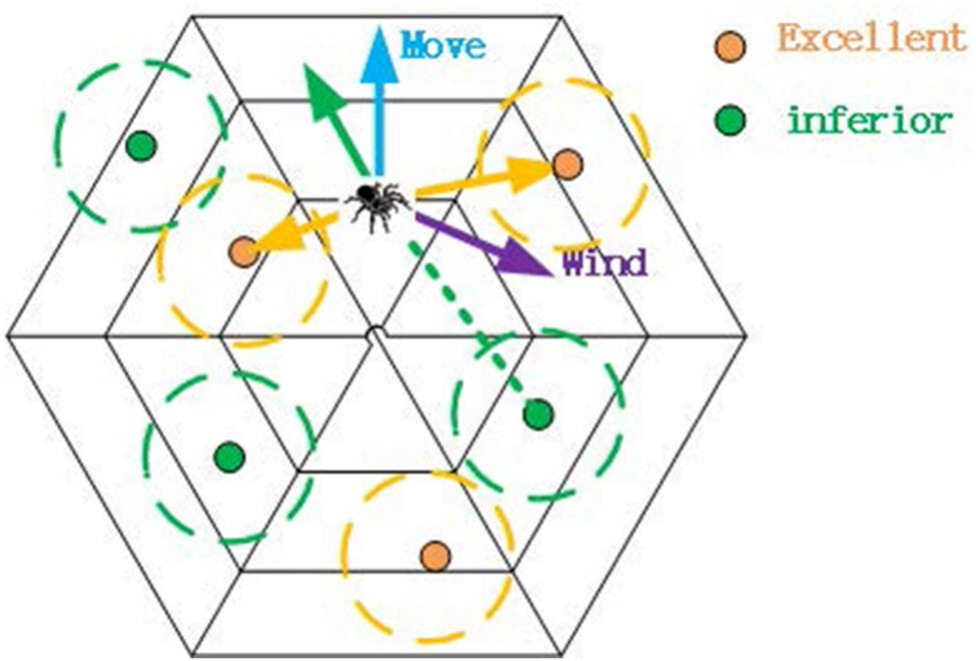
Hunting stage.

These two types of extreme solutions guide the movements of spiders so as not to waste known information resources. Moreover, spiders affected by two kinds of pheromones can catch prey more accurately and exploit the solution space better than spiders affected by only one kind of pheromones. Although the amount of information is multiplied, the base is small, only 2*Y*. So the spiders can still react quickly without affecting the overall speed of iteration. The process in the hunting stage is shown in Fig. 2.

The radius *R* is expressed by the following formula:

7 $$\begin{aligned} R=\frac{\left( ub-lb \right) }{Y}+0.25 \end{aligned}$$

*ub* and *lb* are *D*-dimensional vectors, representing the boundary value of the cobweb in every dimension. *ub* represents the upper bound of *D*-dimensional optimizing space, and *lb* represents the lower bound of *D*-dimensional optimizing space.

The formula for quantifying the effect $W{{g}_{s}}i$ of the extremely good solution ${{g}_{s}}$ on spider *i* is as follows:

8 $$\begin{aligned} w{{g}_{s}}i=\left\{ \begin{matrix} f{{g}_{s}}\times \left( {{g}_{s}}-{{x}_{i}} \right) ,\root D \of {\sum \limits _{k=1}^{D}{\left( {{x}_{ik}}-{{g}_{sk}} \right) }}\le R \\ 0 \,,\root D \of {\sum \limits _{k=1}^{D}{{{\left( {{x}_{ik}}-{{g}_{sk}} \right) }^{2}}}}>R \\ \end{matrix} \right. \end{aligned}$$

The formula for quantifying the total effect $W{{g}_{i}}$ of extremely good solutions on spider *i* is as follows:

9 $$\begin{aligned} Wgi=\sum \limits _{s=1}^{Y}{w{{g}_{s}}i} \end{aligned}$$

Accordingly, the formula for quantifying the effect $W{{b}_{s}}i$ of the extremely bad solution ${{b}_{s}}$ on spider *i* is as follows:

10 $$\begin{aligned} w{{b}_{s}}i=\left\{ \begin{matrix} f{{b}_{s}}\times \left( {{b}_{s}}-{{x}_{i}} \right) ,\root D \of {\sum \limits _{k=1}^{D}{\left( {{x}_{ik}}-{{b}_{sk}} \right) }}\le R \\ 0 \,,\root D \of {\sum \limits _{k=1}^{D}{{{\left( {{x}_{ik}}-{{b}_{sk}} \right) }^{2}}}}>R \\ \end{matrix} \right. \end{aligned}$$

The formula for quantifying the total effect $W{{b}_{i}}$ of extremely bad solutions on spider *i* is as follows:

11 $$\begin{aligned} W{{b}_{i}}=\sum \limits _{s=1}^{Y}{w}{{b}_{s}}i \end{aligned}$$

The formula to quantify the effect ${{W}_{i}}$ of all extreme solutions on spiders *i* is as follows:

12 $$\begin{aligned} {{W}_{i}}=W{{g}_{i}}+W{{b}_{i}} \end{aligned}$$

After the positioning stage of SPC was completed, the weight of spiders in the population increased. Considering that the weight of spiders is an important resistant factor of winds, the effect of winds on movements of spiders should be adjusted. That is to say, at the positioning stage of the algorithm, the winds have a significant effect on the movements of spiders, while at the hunting stage of the algorithm, the effect of winds on spiders should be reduced to some extent. By changing the moving formula, the effect of winds can be reduced appropriately.

The moving step *r* of the spiders at the hunting stage of SPC is expressed by the following formula:

13 $$\begin{aligned} r={{e}^{-h}}\times \left\| ub\times lb \right\| \end{aligned}$$

*h* is the number of iterations at the hunting stage, *ub* and *lb* are *D*-dimensional vectors, representing the boundary value of the cobweb in every dimension. *ub* represents the upper bound of *D*-dimensional optimizing space, and *lb* represents the lower bound of *D*-dimensional optimizing space.

The moving formula of spider *i* at the hunting stage of SPC is as follows:

14 $$\begin{aligned} {{x}_{i}}={{x}_{i}}/{{c}_{m2}}+\left( {{W}_{i}}+wind+{{Z}_{i}} \right) \times r \end{aligned}$$

${{Z}_{i}}$ can be obtained from formula 2, $\text {w}ind$ can be obtained from formula 3, ${{W}_{i}}$ can be obtained from formula 4, *r* can be obtained from formula 3. ${{c}_{m2}}$ is the inertial weight of the hunting stage, it is set to a constant.

If the spider *i* goes beyond the boundary of the cobweb in the process of random walk, the random method is used to deal with the constraint^32^.

### Implement of SPC

By simulating the collaborative hunting process of spiders, the optimizing model of SPC is acquired. SPC has two stages: the interactions of spiders using pheromones and the cobweb’s guidance to spiders’ movements. At the positioning stage, every spider adjusts its location according to the distribution of pheromones on the web. The more the preys at the location are, the more concentration of aggregation pheromones released by the spiders at the location. And the other spiders tend to move to that location in the next movement. On the contrary, the smaller the prey at the location are, the higher the concentration of alarm pheromones released by the spiders are, and the other spiders move away from that location in the next movement. In this way, more feasible solutions can be found to a greater extent, which is helpful to record the extreme solutions for cobweb that can better reflect the global information and make the cobweb guide the movements of the spiders at the hunting stage from the global perspective. At the hunting stage of SPC, spiders communicate with other spiders to produce better solutions by using the mark pheromones and trail pheromones on the web, and the aggregation pheromones and alarm pheromones produced by themselves.

All spiders are equally based on shared information. There is no leader, so there is no case where the individual dominates the movements of the group, but the interactions between spiders guide the movements of the spiders. In this way, it avoids falling into individualism, which results in premature accumulation of the whole population stagnation of evolution. Moreover, the diversity of the population can not be effectively maintained, which is easy to fall into local optimization.

SPC algorithm can jump out of local optimization to realize global optimization because of randomness. Under the adjustment of positive and negative feedback of pheromones, it can not only receive the effect of outstanding solutions but also suppress the interference of horrible solutions. SPC makes full use of the adequate existing information on the cobweb. It does not waste information resources or generate information redundancy.

The new locations of all spiders are constructed by the current locations and historical locations of other spiders in the population. It can retain the past information, prevent the algorithm from jumping too fast, and make the algorithm evolve smoothly. With the increase of the number of iterations, the dynamic moving step *r* decreases nonlinear, which plays a great role in the convergence of SPC. It makes SPC have strong global optimization ability in the positioning stage, at the hunting stage, it gradually converges to a better area. Therefore, it balances the ability of global optimization and local optimization more effectively. In addition, the random vector *wind* can ensure the diversity of the population of spiders and the randomness of SPC algorithm.

SPC assumes that overall optimization space is a cobweb, and spiders can interact indirectly through pheromones. Every solution in the optimization space is represented by a location on the web. The process of SPC algorithm can be described by algorithm 1.

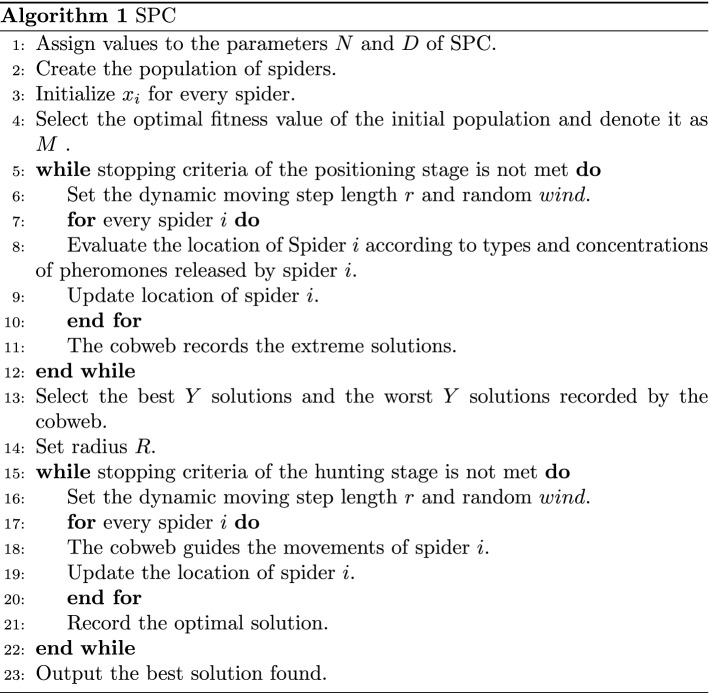



*Step 1* Set the size of population to *N* and the dimension of optimization space *D* , randomly initialize the locations of the population of spiders.

*Step 2* Sort and compare the fitness values of spiders in the initial population, select the spider with the best fitness value, and record its fitness value as *M* .

*Step 3* Spiders interact through pheromones according to formula 1.

*Step 4* The locations and pheromones of spiders were updated respectively according to formula 6.

*Step 5* If the new locations of the spiders exceeds the boundary of the cobweb, the random method is used to constrain the spiders.

*Step 6* If the maximum number of iterations in the positioning phase is not reached, go to step 3.

*Step 7* Select the extreme solutions and record their information.

*Step 8* The cobweb guides the movements of spiders according to formula 8 and formula 10.

*Step 9* Update the locations of the spiders according to formula 14.

*Step 10* If the new locations of the spiders exceeds the boundary of the cobweb, the random method is used to constrain the spiders.

*Step 11* If the maximum number of iterations in the hunting phase is not reached, go to step 8.

### Differences of SPC, SSO and SSA

In SSO, the searching individuals are classified by sex. Male spiders and female spiders have different optimizing operations. SSO simulates the mating behaviors of social spiders.

In SSA, when a spider finds its prey, it creates a strong vibration that attracts the rest of the spiders to move towards it. Eventually, most of the spiders in the group will gather around the prey.

For SSO and SSA, pheromones were not used, and the effect of winds on movements of spiders was not considered.

SPC considers that pheromones of spiders are the communicating medium between organisms. It divides the whole optimization process into two stages. At the positioning stage, the releasing and receiving process of aggregation pheromones and alarm pheromones are abstracted into the process of interactions of spiders. Communications of pheromones complete the update of spiders’ locations. The new locations of spiders are constructed by their historical locations together with the distribution of other spiders and the winds at that time. The primary task of the positioning stage is to explore the solution space as much as possible. SPC gives the cobweb a certain intelligence – the memory so that the cobweb can record the extreme solutions that reflect the global information. At the hunting stage, the movements of spiders are guided by the distribution of the extreme solutions recorded by the cobweb so that the spiders can converge to the good solutions as much as possible. While many intelligent algorithms try to avoid extreme individuals from affecting the optimization process, SPC skillfully uses extreme solutions to guide the movements of the spiders.

## Experiment and result

In this section, the SPC method is evaluated from several aspects. The parameter of SPC would be discussed, and the comparison between SPC and some existing algorithms would be made using a series of benchmarks. The experiments are performed using Matlab R2018a on a Windows laptop with an Intel Core i5 processor and 24 GB of RAM.

### Parameter selection

Choosing appropriate parameters of SPC for numerical and real-world optimization problems can be time-consuming. In this paper, single-factor sensitivity analysis is used to select appropriate parameters. The five parameters in the equations of SPC are further discussed.

${{c}_{w}}$,${{c}_{f}}$: These two parameters jointly determine the effect of wind on the spiders’ movements.

${{c}_{r}}$: This parameter determines the size of the spiders’ moving step.

${{c}_{m1}}$: This parameter determines the effect of the original position of the positioning stage spider on the new location of the spider.

${{c}_{m2}}$: This parameter determines the effect of the original position of the hunting stage spider on the new location of the spider.

**Figure 3:**
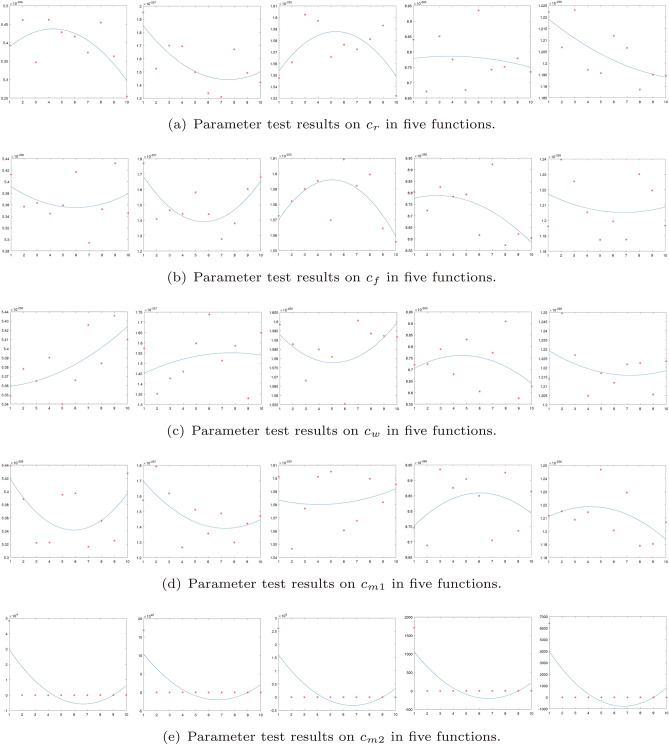
Parameter test results on ${{c}_{r}}$, ${{c}_{f}}$, ${{c}_{w}}$, ${{c}_{m1}}$ and ${{c}_{m2}}$.

In this section, five 30-dimensional benchmark functions are employed to discuss the impact of these parameters on the performance of SPC (these functions are included in supplementaries). The values of ${{c}_{f}}$, ${{c}_{w}}$ and ${{c}_{r}}$ are selected from the set $\left\{ 1,2,3,4,5,6,7,8,9,\left. 10 \right\} \right.$, and the values of ${{c}_{m1}}$ and ${{c}_{m2}}$ are both selected from the set $\left\{ 1,1.5,2,2.5,3,3.5,4,4.5,5,\left. 5.5 \right\} \right.$. For each function/parameter pair there are 10 data points for analysis. The stopping criteria is set to 1000, each function is tested 10 times. The population size of spiders is set to 4 in accordance with our later simulation. The mean values are plotted in Fig. 3 with dots, and the second-order polynomial regression curve for each function is also plotted for demonstration. Some interesting observations can be obtained from the mean results and the regression curve.

From Fig. 3, it can be observed that the experimental results have differences when the four parameters of ${{c}_{r}}$, ${{c}_{f}}$, ${{c}_{w}}$ and ${{c}_{m1}}$ change in the five functions. Therefore, the values of these parameters depend largely on the nature of the optimization problem to be solved. For the parameter ${{c}_{m2}}$, the experimental results are robust.

So the parameters are set as ${{c}_{r}}=2$,${{c}_{f}}=5$,${{c}_{w}}=5$,${{c}_{m1}}=2$ and ${{c}_{m2}}=2.5$ in the next simulation experiment. Please note that this parameter combination can not guarantee the optimal solution for solving all optimization problems . And parameter tuning is essential to address unfamiliar problems.The parameter sensitivity analysis in this paper is preliminary, that is, only one parameter is tested while the remaining four are unchanged.

### Statistical and convergence comparison on 35 benchmarks

Thirty-five standard benchmark functions are used for performance experiments of SPC. These functions are given in supplementary. Some typical or recently proposed algorithms are involved as comparisons, including ABC, ACO, GWO, HHO, MBO, PSO, SSO, and SSA. The key role in improving the performance and efficiency of the algorithm is the choice of values of parameters. For ABC, the number of bees is set to 50. The number of spiders in SPC is set to 4. For ACO, the parameter PF is set to 0.5 , and the number of ants is set to 30. For GWO, the number of gray wolves is set to 10. For HHO, the number of harris hawks is set to 30. For MBO, the population size is set to 50. For PSO, the learning factors ${{c}_{1}}$ and ${{c}_{2}}$ are equally set to 3, the inertia weight $\omega$ is set to 0.5, and particle amount is set to 30. For SSO, the number of spiders is set to 30. For SSA, the number of spiders is set to 25. Other parameters involved in the comparison algorithms are given by their original literature.

In these experiments, the number of iterations is set to 1000. The dimension *D* is unified to 30. The scope of the optimization space is given by the scope of the benchmark functions. Considering the randomness of the parameters in the swarm intelligence algorithm, the experiment of every benchmark function runs 100 times independently. The average values of the convergence results of 100 runs are calculated as the final results. Table 1 lists the statistical results of the algorithms running 100 times independently for 35 benchmark functions. AVG stands for average values, and STD stands for the standard deviation of values. The best results are shown in bold.

**Table 1:** Test results of 35 benchmark functions.

	SPC	SSA	SSO	ABC	ACO	GWO	HHO	MBO	PSO
**fun1**									
AVG	**5.357E–256**	1.487E+00	1.609E+01	3.740E–02	5.351E+00	9.100E–35	2.596E–177	1.959E+04	9.113E–01
STD	0.000E+00	4.397E–01	5.644E+01	2.940E–02	7.879E+00	2.967E–34	0.000E+00	2.435E+04	3.731E–01
**fun2**									
AVG	**1.523E–257**	1.325E+45	4.773E+25	2.201E+00	1.265E+42	5.773E–93	2.888E–227	3.205E+46	1.039E–04
STD	0.000E+00	4.785E+45	4.773E+26	5.765E+00	3.623E+42	5.383E–92	0.000E+00	2.218E+47	1.684E–04
**fun3**									
AVG	**1.577E–255**	6.951E+00	1.863E+02	1.615E–01	2.858e+05	2.342E–34	1.629E–177	3.532E+04	1.042E+01
STD	0.000E+00	3.075E+00	1.498E+03	1.386E–01	3.698e+04	4.577E–34	0.000E+00	8.640E+04	4.042E+00
**fun4**									
AVG	**8.726E–260**	5.990E–02	1.554E–01	3.911E–04	1.579E+00	4.991E–37	2.928E–180	1.820E+03	1.118E+01
STD	0.000E+00	2.610E–02	7.005E–01	3.185E–04	1.584E+00	1.725E–36	0.000E+00	8.668E+02	4.267E+00
**fun5**									
AVG	**1.203E–258**	2.271E–01	1.067E+00	4.100E–03	6.544E+00	9.752E–36	2.748E–186	5.982E+03	1.094E+01
STD	0.000E+00	8.650E–02	3.359E+00	3.000E–03	7.457E+00	2.346E–35	0.000E+00	3.745E+03	4.997E+00
**fun6**									
AVG	**1.655E–253**	1.137E+04	1.556E+03	5.822E+04	5.698E+04	5.655E–06	1.215E–143	5.631E+04	1.576E+00
STD	0.000E+00	2.345E+03	3.746E+03	1.038E+04	6.334E+03	1.656E–05	1.215E–142	3.739E+04	6.611E–01
**fun7**									
AVG	**− 1.000E+00**	− 4.630E–02	− 9.999E–01	− 1.000E+00	− 4.318E–01	− 1.000E+00	**− 1.000E+00**	− 4.443E–01	− 6.454E–01
STD	0.000E+00	1.630E–02	3.229E–04	7.920E–07	7.220E–02	7.231E–17	0.000E+00	4.467E–01	9.730E–02
**fun8**									
AVG	**4.512E–251**	1.444E+03	6.683E+05	6.080E+01	5.845E+08	2.355E–31	3.046E–176	5.494E+08	5.154E+03
STD	0.000E+00	5.846E+02	2.895E+06	3.454E+01	1.206E+08	7.069E–31	0.000E+00	4.000E+08	3.422E+03
**fun9**									
AVG	5.142E–01	4.620E–02	4.190E–02	1.642E–01	3.210E+01	2.100E–03	**7.150E–05**	1.519E+02	1.447E+00
STD	2.935E–01	1.310E–02	3.760E–02	4.180E–02	7.994E+00	1.300E–03	6.831E–05	9.416E+01	7.766E–01
**fun10**									
AVG	**5.312E–129**	3.440E+01	5.722E–01	5.433E+01	8.646E+01	2.829E–08	1.233E–90	3.108E+01	9.961E–01
STD	2.146E–130	4.138E+00	1.162E+00	3.676E+00	3.131E+00	6.183E–08	1.203E–89	2.435E+01	3.930E–02
**fun11**									
AVG	**1.516E–129**	5.578E–01	2.943E–01	5.749E+00	4.327E+04	5.753E–21	9.166E–95	6.940E+09	4.645E+00
STD	2.165E–131	3.142E–01	6.330E–01	1.650E+01	1.606E+05	8.946E–21	8.144E–94	6.313E+10	9.059E–01
**fun12**									
AVG	7.500E+00	1.578E+00	6.185E+00	4.200E–02	5.287E+04	1.688E+00	**3.984E–05**	1.804E+04	1.757E+00
STD	0.000E+00	8.580E–01	2.071E+01	3.300E–02	6.974E+03	4.937E–01	6.052E–05	2.281E+04	7.144E–01
**fun13**									
AVG	**0.000E+00**	9.718E–01	8.515E–01	4.211E–01	4.823E+02	4.200E–03	**0.000E+00**	1.891E+02	3.750E–02
STD	0.000E+00	6.690E–02	1.613E+00	1.711E–01	5.862E+01	9.700E–03	0.000E+00	2.040E+02	1.170E–02
**fun14**									
AVG	− 2.792E+02	2.127E+04	1.060E+03	2.403E+04	2.287E+06	− 1.064E+02	**− 4.206E+03**	1.510E+05	− 5.502E+02
STD	6.537E+01	6.204E+00	1.711E+03	1.388E+04	3.589E+05	9.331E+01	1.347E+02	3.637E+05	1.758E+02
**fun15**									
AVG	**0.000E+00**	4.600E+01	3.819E+00	2.208E+02	2.085E+02	1.865E+00	**0.000E+00**	2.214E+02	2.321E+01
STD	0.000E+00	6.420E+00	9.397E+00	1.422E+01	2.189E+01	3.731E+00	0.000E+00	1.529E+02	5.639E+00
**fun16**									
AVG	1.345E+00	6.100E–03	8.400E–03	1.708E+00	2.694E+01	1.567E+00	**1.121E–05**	1.754E+01	5.190E–01
STD	1.810E–01	2.700E–03	3.140E–02	8.128E–01	4.238E+00	2.706E–01	1.936E–05	2.268E+01	1.557E–01
**fun17**									
AVG	**8.881E–16**	7.272E–01	8.587E–01	1.384E+00	2.059E+01	3.907E–14	**8.881E–16**	1.434E+01	2.683E+00
STD	0.000E+00	2.208E–01	1.336E+00	5.757E–01	1.576E–01	4.354E–15	0.000E+00	7.276E+00	2.763E–01
**fun18**									
AVG	2.871E+01	1.775E+02	2.710E+00	1.468E+03	1.774E+06	2.761E+01	**2.200E–03**	6.984E+05	1.226E+02
STD	6.193E–03	3.452E+01	7.457E+00	8.282E+02	3.366E+05	7.978E–01	2.900E–03	1.106E+06	3.599E+01
**fun19**									
AVG	**4.431E–257**	2.351E+02	3.475E+00	1.432E+03	3.869E+02	8.400E–03	1.510E–72	2.490E+03	2.070E+00
STD	0.000E+00	5.114E+01	1.483E+01	5.492E+02	4.713E+01	7.200E–02	1.510E–71	1.331E+04	7.409E–01
**fun20**									
AVG	9.973E–01	1.216E+01	5.181E–01	9.791E+01	9.366E+05	6.667E–01	**2.493E–01**	1.025E+06	1.130E+01
STD	7.867E–04	3.314E+00	9.471E–01	6.315E+01	1.803E+05	8.852E–06	8.747E–04	7.632E+05	3.853E+00
**fun21**									
AVG	− 8.754E+00	**− 8.693E+00**	− 3.242E+00	− 9.588E+00	− 1.534E+01	− 1.184E+01	− 1.177E+01	− 1.511E+01	− 9.673E+00
STD	6.003E–01	7.107E–01	4.259E+00	5.658E–01	9.176E–01	3.100E+00	1.418E+00	3.164E+00	1.526E+00
**fun22**									
AVG	**2.14E–258**	1.011E+02	7.851E+00	1.246E+04	4.300E+04	8.057E–06	1.114E–182	4.438E+04	5.790E+00
STD	0.000E+00	3.813E+01	7.126E+01	3.864E+03	7.038E+03	1.304E–05	0.000E+00	4.583E+04	4.682E+00
**fun23**									
AVG	**7.646E–254**	4.298E+00	9.682E+00	3.056E+02	4.730E+08	4.321E–31	6.754E–173	4.331E+08	7.736E+05
STD	0.000E+00	1.672E+00	3.710E+00	2.121E+02	4.634E+07	9.889E–31	0.000E+00	2.351E+08	2.845E+05
**fun24**									
AVG	**1.513E–130**	1.210E+00	3.310E–02	2.407E+01	3.432E+01	1.949E–04	1.969E–06	1.763E+01	1.069E+00
STD	1.970E–132	4.448E–01	7.070E–02	2.847E+00	4.078E+00	3.812E–04	1.607E–05	2.232E+01	4.600E–01
**fun25**									
AVG	**0.000E+00**	9.938E+00	1.353E+00	1.994E+01	1.506E+01	5.522E+00	**0.000E+00**	1.160E+01	5.408E+00
STD	0.000E+00	5.786E–01	9.331E–01	5.857E–01	1.145E+00	3.490E+00	0.000E+00	6.835E+00	1.420E+00
**fun26**									
AVG	6.841E+02	7.808E+00	1.034E+00	4.827E+02	6.861E+03	3.860E+02	**− 3.000E–03**	2.727E+03	3.112E+02
STD	4.100E+01	6.540E+00	5.136E+00	2.939E+01	1.424E+03	6.385E+01	3.600E–03	4.780E+03	6.595E+01
**fun27**									
AVG	**5.354E–257**	5.577E–01	1.119E+00	5.176E–01	5.456E+03	3.900E–03	1.199E–170	2.350E+03	2.084E–01
STD	0.000E+00	1.101E–01	3.114E+00	8.560E–02	7.220E+02	4.900E–03	0.000E+00	2.539E+03	6.080E–02
**fun28**									
AVG	**2.307E–63**	4.831E+01	3.053E+00	5.479E+01	3.192E+02	9.581E–09	4.603E–49	1.998E+02	2.506E+01
STD	1.503E–65	5.284E+00	2.635E+00	7.043E+00	1.465E+01	7.164E–09	1.376E–48	9.969E+01	2.857E+00
**fun29**									
AVG	**0.000E+00**	8.717E+00	3.539E+00	1.312E+01	1.407E+01	8.003E+00	**0.000E+00**	1.049E+01	1.174E+01
STD	0.000E+00	4.561E–01	3.028E+00	2.006E–01	4.486E–01	1.525E+00	0.000E+00	3.826E+00	2.154E+00
**fun30**									
AVG	1.668E+00	4.120E–02	4.570E–01	8.355E+04	3.345E+08	1.079E–01	**1.317E–06**	1.174E+08	6.300E–02
STD	8.926E–16	3.480E–02	1.923E+00	1.072E+05	9.446E+07	7.330E–02	1.808E–06	2.012E+08	4.240E–02
**fun31**									
AVG	**− 2.900E+01**	− 5.230E+00	− 9.965E–01	− 6.107E+00	− 9.279E+00	− 2.048E+01	**− 2.900E+01**	− 1.019E+01	− 2.008E+01
STD	0.000E+00	6.620E–01	7.800E–03	5.760E–01	7.583E–01	4.226E+00	0.000E+00	5.850E+00	6.182E–01
**fun32**									
AVG	**0.000E+00**	3.407E+00	9.495E–07	5.392E+00	3.236E+00	4.839E+00	1.037E–10	1.126E+00	6.120E–02
STD	0.000E+00	2.480E–01	4.421E–06	1.492E–01	2.113E–01	2.825E–01	3.009E–10	1.425E+00	7.720E–02
**fun33**									
AVG	**0.000E+00**	3.0041E+01	3.041E+00	2.025E+02	2.712E+02	6.285E+00	**0.000E+00**	1.717E+02	2.428E+01
STD	0.000E+00	3.587E+00	7.029E+00	1.677E+01	2.342E+01	4.570E+00	0.000E+00	1.437E+02	5.837E+00
**fun34**									
AVG	4.139E+02	3.206E+17	1.446E+00	1.966E+15	5.819E+17	4.030E+02	**1.289E–04**	1.136E+17	7.639E+02
STD	2.285E–13	1.305E+17	1.398E+00	1.426E+15	2.050E+17	7.990E+00	4.169E–04	2.469E+17	5.435E+01
**fun35**									
AVG	**7.681E–65**	1.683E+00	8.915E–01	1.920E+00	1.070E+01	2.677E–10	9.746E–49	6.968E+00	8.426E–01
STD	6.415E–67	2.023E–01	7.395E–01	2.590E–01	5.063E–01	1.948E–10	6.235E–48	2.800E+00	9.320E–02

Here are some zeros in Table 1. This is because the values are very close to zero and are smaller than the minimum value that Matlab could discriminate.

From Table 1, the following information could be attained. SPC has the best results on 21 out of 35 benchmark functions, which hold first place on the number of best results. The second most at the number of best results is HHO, which has 16 out of 35 benchmark functions. On some benchmark functions, both SPC and HHO get the best results. SSO has one best results on the experiments. The rest of the algorithms do not take any place in experiments. The results of experiments show the effectiveness and good ability of SPC. The average and standard deviation values from SPC on most of the benchmark functions indicate the accuracy and stability of SPC.

Because unimodal functions have only one global optimal value, they can be used to evaluate the function’s exploitation capability. According to Table 1, SPC has excellent exploitation capability. This can be seen from the optimization results of the SPC for the single peak function. For example, for the unimodal functions fun1 and fun11, the results of SPC are superior to other algorithms, indicating that the SPC has strong competitiveness.

Unlike one-dimensional functions, multimodal functions have many locally optimal solutions, the number of which grows exponentially with the dimension of the problem. Therefore, multimodal functions are very effective for evaluating the exploration capability of the algorithm. Particularly, fun29 is the Schaffer function, which is a two-dimensional complex function with an infinite number of minima. It gets the global minimum of 0 at (0,0). Because the function has strong oscillatory behavior, it is difficult to find the optimal global value in the traditional optimization algorithm. Since SPC explored a wide range of globally feasible solutions during the positioning stage, its results almost approach to 0 completely. In addition, fun13 is the Griewank function, which is a typical nonlinear multimodal function with a broad search space and is generally considered as a complex multimodal problem that is difficult to deal with for optimization algorithms. Its global minimum is obtained at zero, which is 0. SPC also performs very well in the optimization results of this function. Its results are also nearly close to 0, which is obviously better than other algorithms, indicating that its global search ability is better than other algorithms.

The stability of algorithms greatly influences not only the standard deviation but also the average. The reason is that the average value of many executions instead of the medium value that is used as the final result in most cases. For example, assuming the solution of a problem is 0, the final result is the average of the results of 100 executions. The final result would be enormous if the algorithm got zero by 99 out of 100 executions, and only one execution got an enormous value. From Table 1, the results show that SPC is the most stable algorithm and MBO is the most unstable algorithm.

It should be noticed that the number of spiders in SPC is only 4, or that is to say, the search agent of SPC is 4. Compared with the number of search agents, which is 10, 30, or more, SPC uses fewer agents to get better performance, which further demonstrates the superiority of design and construction in SPC.

Make a further investigation on the results obtained by SPC and HHO, and it can be found that SPC can get more precise results than HHO when SPC has a good result on the function, while HHO may have good results on more functions than SPC. In other words, SPC can reach better precision while it may not be effective for that many cases, and HHO could be used in more cases, but results are not that precise. The universality of SPC may be a disadvantage of SPC and should be improved in future work.

**Figure 4:**
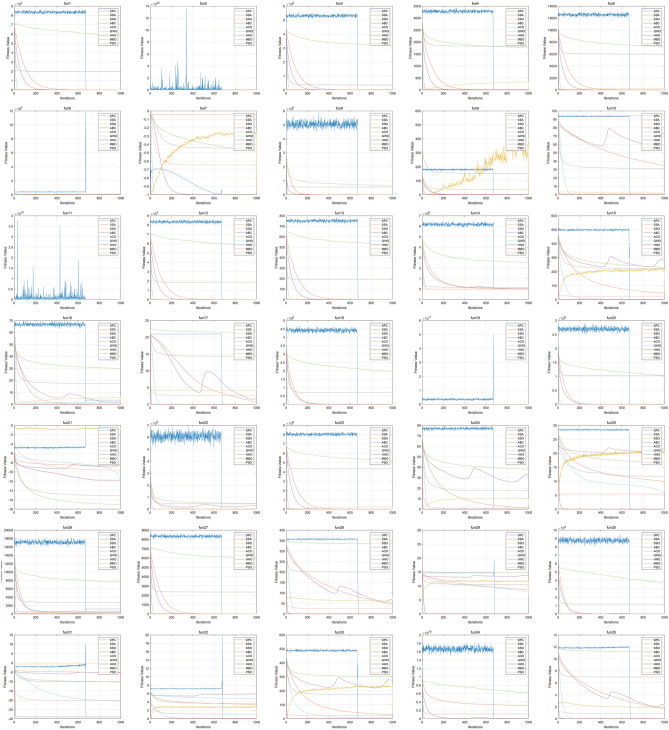
Convergence results of SPC and other algorithms on 35 benchmark functions.

Figure 4 shows the convergence curves of 9 algorithms on 35 benchmark functions. The blue lines stand for the curves of SPC. The convergence curves of SPC obviously reflect the two stages of SPC. During the positioning stage of SPC, it continuously explores feasible solutions in a global scope. SPC will not converge at this stage, so its convergence curve is constantly oscillating at this stage. At the hunting stage, it can quickly converge to the optimal solution of the whole scope, which shows that SPC can escape from the optimal local solution. Some solutions at the global scope may have extreme values, which cause the pulse on convergence curves, like fun2, fun6, fun11, and fun19. While the function is sensitive to the change of solution, the curves of fitness values will move wildly, like fun6 and fun22. SPC is not the most quickly converged algorithm due to the theory of SPC, and through the curve, HHO is the quickest converged algorithm among the algorithms involved.

**Figure 5:**
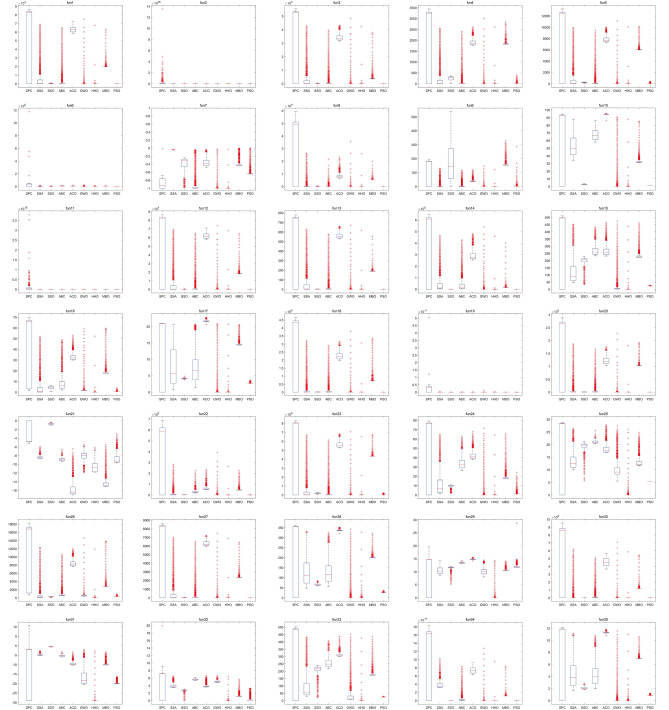
Box plot of SPC and other algorithms on 35 benchmark functions.

Box plot can reflect the spread of data. Figure 5 is the box plot of fitness values during coverage progress for each algorithm on benchmark functions. In Fig. 5, the box of SPC is wide, and the absolute value of the maximum and minimum values of SPC is quite different from other algorithms. Only a few data are close to the optimal global solution, which reflects the structure of the algorithm. The SPC algorithm mainly does not retain the optimal solution during the positioning phase, and it keeps exploring other feasible solutions. That is, the first stage task of SPC is to explore the search space as much as possible so that it will fluctuate in the region of the optimal local solution. At the hunting stage, based on the information obtained in the positioning stage, it can quickly converge to the complete optimal solution, indicating that the improved algorithm has the ability to escape from the optimal local solution.

### Statistical comparison on higher dimensions

From the previous experiments, SPC performs well when the dimension of the problem is 30. To further investigate the performance of SPC, the experiments should be done with problems having higher dimensions. In this case, the dimension of benchmark functions increased to 100, and 6 algorithms are involved in the experiments, including SPC, SSA, SSO, GWO, HHO, and PSO. The results are shown in Table 2.

**Table 2:** Test results of 35 benchmark functions with 100 dimensions.

	SPC	SSA	SSO	GWO	HHO	PSO
**fun1**						
AVG	**6.454E−256**	5.474E+02	8.511E+01	3.782E−18	8.654E−183	6.586E+00
STD	0.000E+00	1.006E+02	3.219E+02	4.147E−18	0.000E+00	1.162E+00
**fun2**						
AVG	**5.333E−258**	4.511E+170	1.005E+64	1.824E−47	3.665E−235	2.000E−03
STD	0.000E+00	Inf	5.507E+64	9.968E−47	0.000E+00	6.500E−03
**fun3**						
AVG	**6.157E−255**	1.006E+04	1.479E+03	6.731E−17	3.012E−178	3.109E+02
STD	0.000E+00	1.422E+03	4.611E+03	7.082E−17	0.000E+00	5.461E+01
**fun4**						
AVG	**3.102E−259**	6.147E+01	2.607E+00	1.516E−19	5.072E−185	2.863E+02
STD	0.000E+00	7.797E+00	1.155E+01	1.230E−19	0.000E+00	5.081E+01
**fun5**						
AVG	**4.533E−258**	2.296E+02	1.714E+01	1.588E−18	1.430E−181	3.137E+02
STD	0.000E+00	3.794E+01	6.624E+01	3.434E−18	0.000E+00	4.676E+01
**fun6**						
AVG	**2.142E−252**	2.243E+05	1.163E+04	2.182E+03	4.116E−102	2.522E+01
STD	0.000E+00	2.042E+04	2.248E+04	2.064E+03	2.254E−101	9.750E+00
**fun7**						
AVG	**− 1.000E+00**	− 2.460E−06	− 9.998E−01	− 1.000E+00	**− 1.000E+00**	− 5.120E−02
STD	0.000E+00	1.315E−06	4.500E−03	4.469E−16	0.000E+00	2.810E−02
**fun8**						
AVG	**4.760E−251**	1.965E+06	1.612E+06	6.108E−15	6.336E−179	1.553E+05
STD	0.000E+00	4.652E+05	2.210E+06	7.629E−15	0.000E+00	5.232E+04
**fun9**						
AVG	5.174E−01	5.715E−01	5.390E−02	6.500E−03	**1.318E−04**	5.133E+01
STD	2.970E−01	9.910E−02	4.590E−02	2.700E−03	1.300E−04	2.090E+01
**fun10**						
AVG	**3.239E−129**	6.396E+01	5.856E−01	1.016E+00	4.326E−93	1.000E+00
STD	1.024E−130	3.121E+00	1.107E+00	9.262E−01	1.347E−92	0.000E+00
**fun11**						
AVG	**2.965E−129**	1.406E+42	8.381E−01	2.411E−11	1.750E−94	2.150E+01
STD	2.060E−131	5.064E+42	1.369E+00	1.050E−11	9.332E−94	2.229E+00
**fun12**						
AVG	2.500E+01	5.518E+02	5.895E+01	1.337E+01	**1.579E−04**	1.583E+01
STD	0.000E+00	1.153E+02	1.301E+02	1.190E+00	2.989E−04	2.496E+00
**fun13**						
AVG	**0.000E+00**	6.351E+00	3.584E+00	5.900E−03	**0.000E+00**	1.036E−01
STD	0.000E+00	9.633E−01	7.348E+00	1.300E−02	0.000E+00	2.000E−02
**fun14**						
AVG	− 1.621E+03	2.095E+07	3.708E+04	7.906E+00	**− 3.939E+04**	− 8.199E+02
STD	1.796E+02	2.919E+06	4.187E+04	3.314E+01	2.079E+03	1.249E+02
**fun15**						
AVG	**0.000E+00**	5.465E+02	1.341E+01	4.434E+00	**0.000E+00**	1.015E+02
STD	0.000E+00	2.457E+01	2.660E+01	6.357E+00	0.000E+00	1.274E+01
**fun16**						
AVG	6.838E+00	1.763E+00	5.500E−02	7.249E+00	**3.230E−05**	2.987E+00
STD	5.659E−01	2.782E−01	2.211E−01	4.680E−01	4.658E−05	3.510E−01
**fun17**						
AVG	**8.881E−16**	4.676E+00	1.407E+00	1.745E−10	**8.881E−16**	2.901E+00
STD	0.000E+00	2.204E−01	1.535E+00	9.108E−11	0.000E+00	1.474E−01
**fun18**						
AVG	9.818E+01	2.871E+03	2.661E+00	9.800E+02	**4.700E−03**	7.059E+02
STD	1.510E−02	6.522E+02	8.108E+00	6.917E−01	6.500E−03	1.069E+02
**fun19**						
AVG	**1.6432E−255**	2.671E+03	4.102E+01	3.589E+03	9.743E−29	1.533E+01
STD	0.000E+00	3.746E+02	7.961E+01	2.047E+03	5.335E−28	3.934E+00
**fun20**						
AVG	9.998E−01	1.905E+03	7.269E+00	6.778E−01	**2.507E−01**	3.508E+02
STD	6.568E−05	5.394E+02	3.195E+01	6.080E−02	1.400E−03	8.714E+01
**fun21**						
AVG	− 1.996E+01	− 2.013E+01	− 2.446E+01	− 1.985E+01	− 2.728E+01	**− 1.601E+01**
STD	9.600E−01	8.115E−01	1.331E+01	3.237E+00	2.806E+00	2.540E+00
**fun22**						
AVG	**2.688E−258**	4.520E+03	4.126E+00	4.960E−05	2.420E−186	1.401E+02
STD	0.000E+00	1.175E+03	1.291E+01	8.870E−05	0.000E+00	3.941E+01
**fun23**						
AVG	**8.921E−254**	4.652E+06	8.570E+05	4.191E−14	1.104E−178	6.485E+06
STD	0.000E+00	8.220E+05	2.948E+06	7.344E−14	0.000E+00	1.225E+06
**fun24**						
AVG	**2.968E−130**	3.739E+01	2.854E−01	7.377E−04	5.186E−06	7.677E+00
STD	2.005E−132	4.409E+00	6.189E−01	1.400E−03	2.840E−05	1.509E+00
**fun25**						
AVG	**0.000E+00**	6.255E+01	3.024E+00	1.632E+01	3.270E−02	2.723E+01
STD	0.000E+00	9.664E−01	4.301E−01	6.785E+00	1.793E−01	2.352E+00
**fun26**						
AVG	2.808E+03	8.632E+02	6.356E+00	2.292E+03	**− 9.200E−03**	1.631E+03
STD	3.658E+01	6.679E+01	1.992E+01	1.445E+02	1.010E−02	1.270E+02
**fun27**						
AVG	**6.429E−257**	5.262E+01	8.821E+00	3.600E−03	2.639E−158	1.000E+00
STD	0.000E+00	8.142E+00	1.643E+01	4.700E−03	1.299E−157	1.376E−01
**fun28**						
AVG	**5.956E−63**	4.020E+02	1.238E+02	4.100E−03	1.1107E−48	9.610E+01
STD	2.306E−65	2.170E+01	9.801E+01	5.800E−03	2.738E−48	3.421E+00
**fun29**						
AVG	**0.000E+00**	4.235E+01	1.438E+01	3.560E+01	**0.000E+00**	4.364E+01
STD	0.000E+00	4.485E−01	1.173E+01	4.542E+00	0.000E+00	4.141E+00
**fun30**						
AVG	1.325E+00	8.879E+00	4.471E−01	4.798E−01	**7.751E−07**	1.470E−01
STD	0.000E+00	1.728E+00	9.883E−01	9.670E−02	5.644E−07	3.550E−02
**fun31**						
AVG	**− 9.900E+01**	− 8.888E+00	− 9.949E−01	− 6.022E+02	**− 9.900E+01**	− 6.058E+01
STD	0.000E+00	9.885E−01	1.180E−02	1.401E+01	0.000E+00	1.786E+00
**fun32**						
AVG	**0.000E+00**	1.832E+01	8.629E−06	2.142E+01	3.323E−10	8.002E−01
STD	0.000E+00	3.835E−01	2.808E−05	4.658E−01	1.019E−10	4.883E−01
**fun33**						
AVG	**0.000E+00**	4.943E+02	9.997E+00	1.245E+01	**0.000E+00**	9.448E+01
STD	0.000E+00	2.700E+01	2.082E+01	2.003E+01	0.000E+00	9.842E+00
**fun34**						
AVG	4.599E+03	1.234E+19	2.113E+03	4.726E+03	**3.080E−04**	1.001E+04
STD	9.250E−13	1.701E+18	6.419E+03	1.586E+02	7.263E−04	3.194E+02
**fun35**						
AVG	**5.954E−65**	4.049E+00	1.382E+00	5.055E−05	1.288E−51	9.655E−01
STD	1.935E−67	2.247E−01	9.015E−01	6.882E−05	3.142E−51	5.010E−02

Significant values are in bold.

From Table 2, the results of SPC are close to previous experiments. It can be concluded that the increase of dimensions has nearly no effect on the performance of SPC, both on precision and stability. SPC also performs well on high-dimensional problems. The increase of dimensions negatively affects the other algorithms at different levels. For SSA, the results of some functions get greatly worse, like fun2 from 1.325e+45 for 30 dimensions to 4.511e+170 for 100dimensions, fun11 from 5.578e−01 for 30 dimensions to 1.406e+42 for 100 dimensions and so on. Most of the rest get slightly worse. The situation of SSO is similar to SSA. For GWO and HHO, the influence of the increase of dimension is mainly on the precision of results. Most of the results do not change a lot but are less accurate than that of 30 dimensions. For PSO, most of the results are slightly worse than that of 30 dimensions. It can be said that SPC has excellent performance when the dimensions increase compared to the other algorithms involved in the experiments.

### Comparison on engineer problems

In this part, SPC is applied to two engineering problems: three-bar truss design problem and tension-compression spring design problem. The results of SPC are compared to several algorithms, including GWO, HHO, SSA, SSO, PSO.

**Figure 6:**
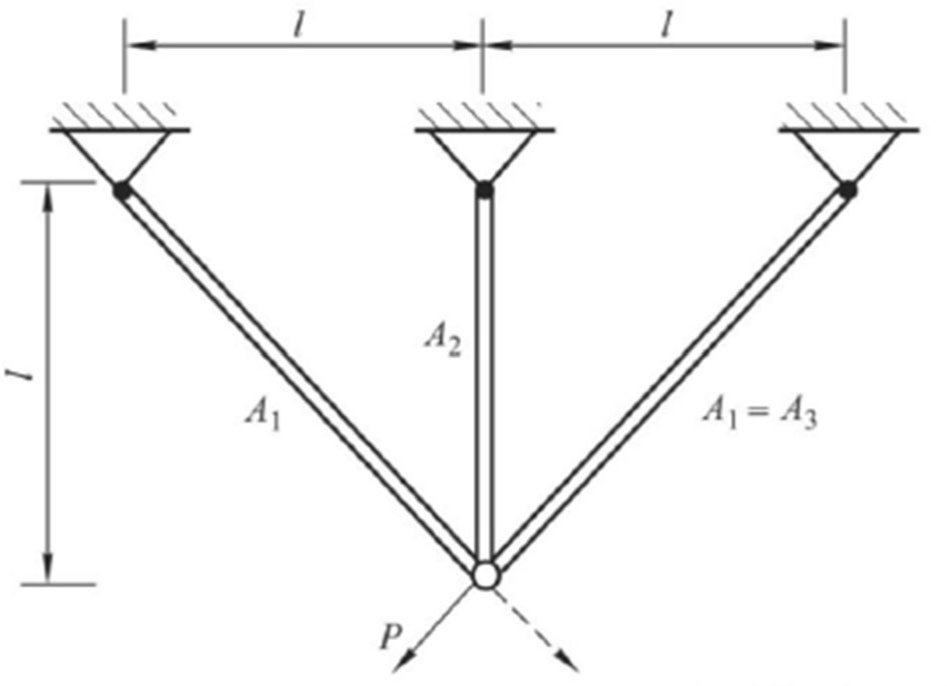
Schematic of the three-bar truss design problem.

The three-bar truss design problem is a well-known structural design problem in practical engineering applications. A graphical illustration of some components of the design problem is shown in Fig. 6. The problem aims to obtain the minimum weight, but it also needs to be subject to several constraints , such as stress, deflection, and buckling constraints. The problem is formulated as follows.

15 $$\begin{aligned} \text {Consider }x=[{{x}_{1}}{{x}_{2}}]=[{{A}_{1}}{{A}_{2}}] \end{aligned}$$

16 $$\begin{aligned} \text {Min f(x)=(2}\sqrt{2}{{x}_{1}}+{{x}_{2}}\text {)}\cdot \text {l} \end{aligned}$$

17 $$\begin{aligned} s.t.\left\{ \begin{matrix}{{g}_{1}}(x)=\frac{\sqrt{2}{{x}_{1}}+{{x}_{2}}}{\sqrt{2}x_{1}^{2}+2{{x}_{1}}{{x}_{2}}}P-\sigma \le 0\\ {{g}_{2}}(x)=\frac{{{x}_{2}}}{\sqrt{2}x_{1}^{2}+2{{x}_{1}}{{x}_{2}}}P-\sigma \le 0\\ \ {{g}_{3}}(x)=\frac{1}{\sqrt{2}{{x}_{2}}+{{x}_{1}}}P-\sigma \le 0 \end{matrix}\right. \end{aligned}$$

The tension-compression spring design problem is another design problem in practical engineering applications. It aims to optimize the weight of the spring. And during the optimization, shear stress, surge frequency, and minimum deflection should be satisfied during the weight optimization. The problem is formulated as follows.

18 $$\begin{aligned} \text {Consider }x=[{{x}_{1}}{{x}_{2}}{{x}_{3}}]=[{{A}_{1}}{{A}_{2}}{{A}_{3}}] \end{aligned}$$

19 $$\begin{aligned} \text {Min f(x)=(}{{x}_{3}}\text {+2)}{{\text {x}}_{\text {2}}}\text {x}_{1}^{2} \end{aligned}$$

20 $$\begin{aligned} s.t.\left\{ \begin{matrix}{{g}_{1}}(x)=1-\frac{x_{2}^{3}{{x}_{3}}}{71785x_{1}^{4}}\le 0 \\ {{g}_{2}}(x)=\frac{4x{}_{2}^{2}-{{z}_{1}}{{z}_{2}}}{12566({{z}_{2}}z_{1}^{3}-z_{1}^{4})}+\frac{1}{5108z_{1}^{2}}\le 0 \\ {{g}_{3}}(x)=1-\frac{140.45{{z}_{1}}}{z_{2}^{2}{{z}_{3}}}\le 0 \\ {{g}_{4}}(x)=\frac{{{z}_{1}}+{{z}_{2}}}{1.5}-1\le 0 \end{matrix}\right. \end{aligned}$$

The results of the experiments are shown in Table 3. In Table 3, three-bar truss design problem is referred to as EF1, and tension-compression spring design problem is referred to as EF2.

**Table 3:** Test results of engineering problems.

	SPC	SSA	SSO	GWO	HHO	PSO
EF1	186.4018	186.3859	186.5081	186.3859	186.3859	186.3859
EF2	3.6706	3.6643	4.5359	3.6619	3.6619	409.7750

The results show that all the involved problems have similar performance on the two engineering problems except PSO on EF2. The reason why they have similar results may be the low dimension and complexity of the problems. And it makes the algorithms can not show their advantages. The experiment results indicate that SPC can also have acceptable performance on typical problems.

### Discussion on results

The parameters of SPC are carefully selected. The performance of the method proposed in this paper, SPC, is tested from three aspects. The results of SPC on most of the 35 benchmark functions show the precision and stability of SPC. And the experiments with higher dimensions indicate that SPC is quite suitable for high dimension problems, the increase of dimension does not negatively affect the performance of SPC. The experiments of typical engineering problems validate its performance on application. With the advantages mentioned, SPC also has problems to be improved. For example, its universality is ferior to some existing algorithms.

## Conclusion

In this work, a novel spider algorithm based on pheromones and cobwebs called SPC is proposed. A positive and negative feedback mechanism in the population of spiders is established by accurately using the effects of different pheromones on spiders. Furthermore, it performs an important role in the progress of SPC. The memory of the cobweb is proposed to be used in the algorithm to realize the communication of pheromones across time. The performance of SPC was investigated. The experiment results show that SPC had excellent performances compared with other algorithms.

In future work, SPC should be further adjusted to obtain better performance, and the weak points of SPC should be strengthened. Besides that, a complete parameter sensitivity analysis is one of the future research topics of SPC. After that, applying this algorithm to practical problems and verifying the performance of the algorithm by extending this algorithm to multi-objective scenarios are the important directions.

## Supplementary Information

Supplementary Information.
