# Supplementary material for: An improved spider optimization algorithm coordinated by pheromones
Source: Sci Rep. 2022 Apr 8;12:5962. doi: 10.1038/s41598-022-09800-x (PMC8993850; doi:10.1038/s41598-022-09800-x)
Supplement: Supplementary file 1 — Supplementary Information. [file 41598_2022_9800_MOESM1_ESM.pdf]

Table A. 35 benchmark functions

Number	Function name	Formula	Bounds	Optimum
fun1	Sphere	$F01=\sum_{i=1}^{d} x_i^2$	$[-100,100]^d$	0
fun2	Sum of different powers	$F02=\sum_{i=1}^{d} \lvert x_i \rvert^{i+1}$	$[-100,100]^d$	0
fun3	Rotated hyper-ellipsoid	$F03=\sum_{i=1}^{d}\sum_{j=1}^{i} x_j^2$	$[-65,65]^d$	0
fun4	Axis parallel hyperellipoid	$F04=\sum_{i=2}^{d} ix_i^2$	$[-5.12,5.12]^d$	0
fun5	Sum Squares	$F05=\sum_{i=1}^{d} ix_i^2$	$[-10,10]^d$	0
fun6	Schwefel's problem 12	$F06=\sum_{i=1}^{d}\left(\sum_{j=1}^{i} x_j\right)^2$	$[-100,100]^d$	0
fun7	Exponential problem	$F07=-\exp\left(-0.5\sum_{i=1}^{d} x_i^2\right)$	$[-1,1]^d$	−1
fun8	High conditioned elliptic	$F08=\sum_{i=1}^{d}\left(10^6\right)^{\frac{i-1}{d-1}} x_i^2$	$[-100,100]^d$	0
fun9	Quartic	$F09=\sum_{i=1}^{d} ix_i^4 + rand(\ )$	$[-1.28,1.28]^d$	0
fun10	Schwefel's problem 2.21	$F10=\max\left\{\lvert x_i \rvert, 1 \le i \le d\right\}$	$[-100,100]^d$	0
fun11	Schwefel's problem 2.22	$F11=\sum_{i=1}^{d}\lvert x_i \rvert + \prod_{i=1}^{d}\lvert x_i \rvert$	$[-10,10]^d$	0
fun12	Step	$F12=\sum_{i=1}^{d}\left(\lvert x_i + 0.5 \rvert\right)^2$	$[-100,100]^d$	0
fun13	Griewank	$F13=\sum_{i=1}^{d}\frac{x_i^2}{4000} - \prod_{i=1}^{d}\cos\left(\frac{x_i}{\sqrt{i}}\right) + 1$	$[-600,600]^d$	0
fun14	Trid	$F14=\sum_{i=1}^{d}(x_i-1)^2 - \sum_{i=2}^{d} x_i x_{i-1}$	$\left[-d^2,d^2\right]^d$	$\frac{-d(d+4)(d-1)}{6}$
fun15	Rastrigin	$F15=10d + \sum_{i=1}^{d}\left[x_i^2 - 10\cos(2\pi x_i)\right]$	$[-5.12,5.12]^d$	0
fun16	Levy	$F16=\sin^2(\pi w_1) + \sum_{i=1}^{d-1}(w_i-1)^2\left[1+10\sin^2(\pi w_i+1)\right] + (w_d-1)^2\left[1+10\sin^2(\pi w_d)\right]$ $where\ \ w_i = 1 + (x_i-1)/4$	$[-5.12,5.12]^d$	0
fun17	Ackley	$F17=-20\exp\left(-0.2\sqrt{\frac{1}{d}\sum_{i=1}^{d} x_i^2}\right) - \exp\left(\frac{1}{d}\sum_{i=1}^{d}\cos(2\pi x_i)\right) + 20 + \exp(1)$	$[-32,32]^d$	0
fun18	Rosenbrock	$F18=\sum_{i=1}^{d-1}\left[100\left(x_{i+1}-x_i^2\right)^2 + (x_i-1)^2\right]$	$[-10,10]^d$	0
fun19	Zakharov	$F19=\sum_{i=1}^{d} x_i^2 + \left(\sum_{i=1}^{d} 0.5ix_i\right)^2 + \left(\sum_{i=1}^{d} 0.5ix_i\right)^4$	$[-5,10]^d$	0
fun20	Dixon-price	$F20=(x_1-1)^2 + \sum_{i=2}^{d} i\left(2x_i^2 - x_{i-1}\right)^2$	$[-10,10]^d$	0
fun21	Michalewicz	$F21=-\sum_{i=1}^{d}\sin(x_i)\sin^{20}\left(\frac{ix_i^2}{\pi}\right)$	$[0,\pi]^d$	0
fun22	Powell	$F22=\sum_{i=1}^{d/4}\left[\left(x_{4i-3}+10x_{4i-2}\right)^2 + 5\left(x_{4i-1}-x_{4i}\right)^2 + \left(x_{4i-2}-2x_{4i-1}\right)^4 + 10\left(x_{4i-3}+x_{4i}\right)^4\right]$	$[-10,10]^d$	0
fun23	Bent cigar	$F23=x_1^2 + 10^6\sum_{i=2}^{d} x_i^2$	$[-10,10]^d$	0
fun24	Alpine	$F24=\sum_{i=1}^{d}\lvert x_i\sin(x_i) + 0.1x_i \rvert$	$[-10,10]^d$	0
fun25	Weierstrass	$F25=\sum_{i=1}^{d}\left(\sum_{k=0}^{20}\left[0.5^k\cos\left(2\pi\cdot 3^k(x_i+0.5)\right)\right]\right) - d\sum_{k=0}^{20}\left[0.5^k\cos\left(2\pi\cdot 3^k\cdot 0.5\right)\right]$	$[-0.9,0.9]^d$	0
fun26	Styblinski-Tang	$F26=0.5\sum_{i=1}^{d}\left(x_i^4 - 16x_i^2 + 5x_i\right) + 39.16599d$	$[-10,10]^d$	0
fun27	Salomon	$F27=1-\cos\left(2\pi\sum_{i=1}^{d} x_i\right) + 0.1\sum_{i=1}^{d} x_i^2$	$[-100,100]^d$	0
fun28	Expansion of F10	$F28=f_{10}(x_1,x_2) + \cdots + f_{10}(x_{i-1},x_i) + f_{10}(x_d,x_1)$ $where\ \ f_{10}(x,y) = \left(x^2+y^2\right)^{0.25}\left[\sin^2\left(50\left(x^2+y^2\right)^{0.1}\right)+1\right]$	$[-100,100]^d$	0

fun29	Expansion Scaffer	$F29 = f_s(x_1,x_2) + f_s(x_2,x_3) + \cdots + f_s(x_d,x_1)$<br>$where\ \ f_s(x,y) = 0.5 + \frac{\sin^2\left(\sqrt{x^2+y^2}\right) - 0.5}{\left(1+0.001\left(x^2+y^2\right)\right)^2}$	$[-100,100]^d$	0
fun30	Generalized penalized	$F30 = \frac{\pi}{d}\left\{10\sin^2(\pi y_1) + \sum_{i=1}^{d-1}(y_i-1)^2\left[1+10\sin^2(\pi y_{i+1})\right] + (y_d-1)^2\right\} + \sum_{i=1}^{d}\mu(x_i,10,100,4)$<br>$where\ \ y_i = 1+\frac{1}{4}(x_i+1), \mu(x_i,a,k,m) = \begin{cases} k(x_i-a)^m, & x_i > a \\ 0, & -a \le x_i \le a \\ k(-x_i-a)^m, & x_i < -a \end{cases}$	$[-50,50]^d$	0
fun31	Inverted cosine wave	$F31 = -\sum_{i=1}^{d-1}\left(\exp\left(-\frac{x_i^2+x_{i+1}^2+0.5x_ix_{i+1}}{8}\right)\times\cos\left(4\sqrt{x_i^2+x_{i+1}^2+0.5x_ix_{i+1}}\right)\right)$	$[-5,5]^d$	$1-d$
fun32	Pathologic	$F32 = \sum_{i=1}^{d-1}\left(0.5 + \frac{\sin^2\left(\sqrt{100x_i^2+x_{i+1}^2}\right)-0.5}{1+0.001\left(x_i^2-2x_ix_{i+1}+x_{i+1}^2\right)^2}\right)^2$	$[-100,100]^d$	0
fun33	Non-continuous Rastrigin	$F33 = \sum_{i=1}^{d}\left[y_i^2 - 10\cos(2\pi y_i) + 10\right]$<br>$where\ \ y_i = \begin{cases} x_i & \lvert x_i\rvert < 1/2 \\ round(2x_i)/2 & \lvert x_i\rvert \ge 1/2 \end{cases}$	$[-5.12,5.12]^d$	0
fun34	Whitley	$F34 = \sum_{k=1}^{d}\sum_{j=1}^{d}\left(\frac{y_{jk}^2}{4000} - \cos(y_{jk}) + 1\right)$<br>$where\ \ y_{jk} = 100\left(x_k - x_j^2\right)^2 + \left(1-x_j^2\right)^2$	$[-100,100]^d$	0
fun35	Schaffer F7	$F35 = \frac{1}{d-1}\sum_{i=1}^{d-1}\left[\left(x_i^2+x_{i+1}^2\right)^{0.25} + \left(x_i^2+x_{i+1}^2\right)^{0.25}\sin^2\left(50\left(x_i^2+x_{i+1}^2\right)^{0.1}\right)\right]$	$[-100,100]^d$	0
